# Supplementary material for: Determining minimal clinically important differences in the North Star Ambulatory Assessment (NSAA) for patients with Duchenne muscular dystrophy
Source: PLoS One. 2023 Apr 26;18(4):e0283669. doi: 10.1371/journal.pone.0283669 (PMC10132589; doi:10.1371/journal.pone.0283669)
Supplement: S1 Appendix — (DOCX) [file pone.0283669.s001.docx]

**Appendix 1**

**North Star Ambulatory Assessment (NSAA) - Questions to Parents/Legal guardians, Children & Young People**

Date__/__/_____

Performed by

Thank you for taking the time to answer the following questions. There are no right or wrong answers and the questionnaire is completely anonymous.

I am the (please tick the appropriate box): Mother Father Legal guardians Child/Young Person

Please indicate your/your child’s age ________

Regarding your child’s current mobility select **one** that best applies:

|  | *Select one* |
| --- | --- |
| Walks independently long distances outdoors (more than 1 km) |  |
| Walks independently medium distances outdoors (less than 1 km) |  |
| Walks outdoors for short distances, e.g. to car with or without help from a person |  |
| Walks indoors with or without help from a person but requires wheelchair for outdoors |  |

| Please indicate if in the last year your child | He has remained stable |  |
| --- | --- | --- |
|  | He has experienced a deterioration in motor abilities |  |
|  | He has improved some motor abilities |  |

| What do you expect for your child in the | He will remain stable |  |
| --- | --- | --- |
| coming 2 years? | He will experience a deterioration in motor skills |  |
|  | He will improve some motor abilities |  |

**Question 1:** In your view, which are the most important **physical** activities/functions of daily life that you would like to see **maintained** in your child in the next year? Can you list at least 3 of them?

1) ___________________________________________________________________________

2) ___________________________________________________________________________

3) ___________________________________________________________________________

Please, now take a look at the separate information sheet that you were given:

**Question 2.** Consider the **activities in A**. Would you consider a meaningful change in your child’s daily life if he could **continue to score 1** in:

At least 1 activity

**(Tick one box only)**

At least 2 activities

More than 2 activities

**Question 3.** Consider the **activities in B**. Would you consider a meaningful change in your child’s daily life if he could **continue to score 2** in:

At least 1 activity

**(Tick one box only)**

At least 2 activities

More than 2 activities

**Question 4:** For your child to take part in a clinical trial lasting 2 years, what would be your **minimum requirement** for the outcome of the trial? (tick one box only)

To improve motor function? 🡪 go to **5**

To slow down a possible decline in motor function? 🡪 go to **6**

To completely stop any decline in motor function? 🡪 no more questions to answer

**Question 5:** Considering the **activities in A** would you consider participating in a clinical trial lasting 2 years if your child could:

**Improved from a score 1 to 2 in:**

At least 1 activity

At least 2 activities

More than 2 activities

**(Tick one box only)**

**Question 6:** Considering **activities in B** would you consider participating in a clinical trial lasting 2 years if your child had the possibility of **slowing down the decline in**:

At least 1 activity

**(Tick one box only)**

At least 2 activities

More than 2 activities

Thank you

**North Star Ambulatory Assessment (NSAA) Information Sheet**

NOTE: We will give this as a separate page and we will add individual items for each patient

**NSAA Score: ………./34**

In the North Star Scale that your child usually performs **each item is scored** in a **scale of 0 to 2** as below

1

Able to perform but with difficulties

0

Not able to perform

2

Able to perform without limitations

1. In the most recently done North Star Scale Assessment, these are the 3 activities your child had difficulties in performing (score 1) and for which **a loss** (score 0) **could** be expected in the future:

**A)**

**A)**

**A)**

1. In the most recently done North Star Scale Assessment, these are the 3 activities that your child was able to perform without limitations (score 2) and for which a **deterioration** **but not complete loss** (score 1) could be expected in the future:

**B)**

**B)**

**B)**
