# Supplementary material for: Determining minimal clinically important differences in the North Star Ambulatory Assessment (NSAA) for patients with Duchenne muscular dystrophy
Source: PLoS One. 2023 Apr 26;18(4):e0283669. doi: 10.1371/journal.pone.0283669 (PMC10132589; doi:10.1371/journal.pone.0283669)
Supplement: S2 Appendix — (PDF) [file pone.0283669.s002.pdf]

Appendix 2

NORTH STAR AMBULATORY ASSESSMENT

30th September 2020

North Star Ambulatory Assessment (NSAA) © 2017 Great Ormond Street Hospital NHS Foundation Trust & The Newcastle upon Tyne Hospitals NHS Foundation Trust

The NSAA is copyright © 2017 The Newcastle upon Tyne Hospitals NHS Foundation Trust & Great Ormond Street Hospital NHS Foundation Trust on behalf of the **UK North Star Clinical Network and Muscular Dystrophy UK**. You may print or use the NSAA for academic or research purposes but any modification, translation or reproduction for redistribution or commercial exploitation of part or all of the contents in any form is strictly prohibited without our express written permission.

GOSH Contact: Vandana Gupta: Vandana.AyyarGupta@gosh.nhs.uk
Newcastle Contact: Anna Mayhew: anna.mayhew1@nhs.net

1.1 Test Description

- The NSAA is a 17-item scale that grades performance of various functional skills on a scale from 0 (unable), 1 (completes independently but with modifications), and 2 (completed without compensation).
- NOTE: The order has been modified to enable more efficient conduct of the assessment.
- Climb and Descend stairs: climb box step is performed step up right, step down right, step up left, step down left.
- Lifts head is performed before ‘gets to sitting’ and ‘rise from floor’ to avoid fatigue.

1.2 Test Guidelines

- Performance of upright activities must not be completed if a lower limb injury exists. However, the subject may perform the head lift and gets-to-sitting items if he is able. Provide ‘comments’ on the source worksheet.
- Complete the NSAA in the order provided (Please note this has been modified from previous trials for a smoother administration).
- The patient should be barefoot.
- Do NOT use a mat.
- The timed 10-meter run/walk and timed rise from floor are conducted as part of the NSAA.
- For all timed function tests (10-meter run/walk, climb 4 stairs, descend 4 stairs, rise from floor), the time in seconds is rounded to the nearest tenth of a second (e.g., 5.11 to 5.14 seconds should be rounded down to 5.1 seconds, 5.15 to 5.19 should be rounded up to 5.2 seconds.
- Generally, activities are graded in the following manner:
  - Score of 2 = ‘Normal’ – no obvious modification of activity
  - Score of 1 = Modified method but achieves goal with no physical assistance
  - Score of 0 = Unable to achieve goal independently
- If you think the subject is capable of a better performance, due to non-compliance or improved understanding of task, it is acceptable to ask the subject to repeat the item if appropriate. Only the best attempt will be scored and documented on the worksheet and in the database.
- If unsure if the performance on a particular item meets the higher criteria, give the subject the lower score.

1.3 10m timed run Set-Up

- Mark off a 10-metre course in a long, quiet hallway or room. Stand approximately 1 to 2 meters beyond the finish line to ensure the subject moves ‘through’ the 10-metre line, rather than slowing down prior to finishing.
- Ensure a clear floor space, measuring approximately 10 feet x 12 feet, is available for remaining assessments (i.e., rise from floor).

1.4 Conducting the Test

1.5 ITEM 1: STANDING			
Starting position	Feet must be no further than 10 cm apart and heels on the ground if possible. Arms by sides.		
Instruction	Can you stand up tall for me for as long as you can and as still as you can for three seconds with your heels flat on the ground?		
Scoring detail	Score is based on ability to hold correct posture and placement of the heels. Score is not based on lordosis.<br>When counting to 3 – Use “And 1, and 2, and 3” so that three seconds is achieved on the word of 3.<br>Score 2 - Minimum count of 3 seconds. Hips must be in alignment with shoulders. Feet must also be pointing forwards and not turned out.		
Activity	2	1	0
Standing	Stands upright, still, symmetrical, without compensation (heels flat and hips in neutral rotation) for minimum count of 3 seconds	Stands still but with compensation (e.g. on toes or with legs abducted or with bottom stuck out/hip flexion, etc.) for minimum count of 3 seconds	Cannot stand still or cannot stand independently, needs support (even minimal)
Photographs/<br>Notes	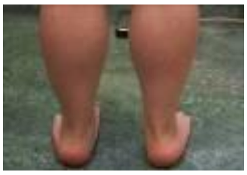<br>Score 2 -Symmetrical heels flat<br>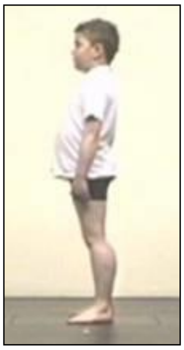<br>Score 2 – Heels down and hips in alignment with shoulders	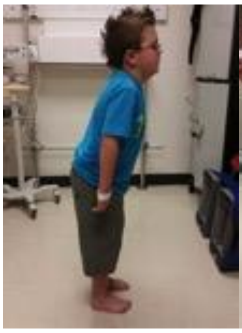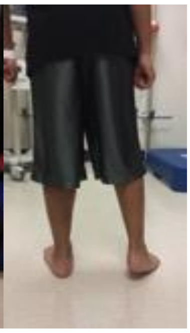<br>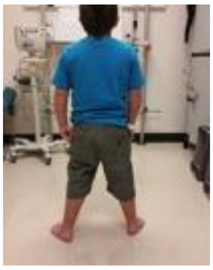<br>Score 1 – Asymmetrical posture or compensation (heels off floor, bottom stuck out)	

1.6 ITEM 2: WALK			
Starting position	Standing. Observe walk for at least 10 steps in both sagittal and coronal planes.		
Instruction	Can you walk from A to B (state to and where from – approximately 8-10 feet) for me?		
Scoring detail	Score is based on ability to place heels down when walking forwards. You do not score them as they are turning around.<br>Score 1 - If one or both heels are off the floor as they step or they need to pause to push the heel down to achieve this.		
Activity	2	1	0
Walk	Walks consistently with heel-toe or flat-footed gait pattern	Persistent or habitual toe walker, unable to heel-toe consistently	Loss of independent ambulation – may use knee-ankle-foot orthosis (KAFO) or walk short distances with assistance
Photographs/<br>Notes	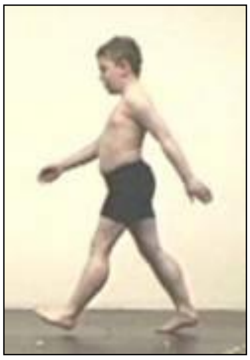<br>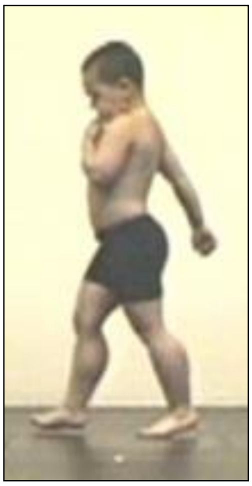	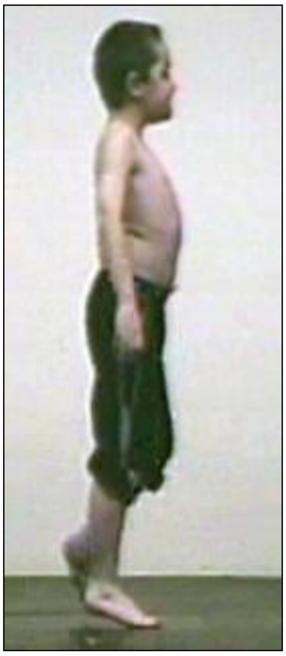<br>Score 1 – habitual toe walker	

1.7 ITEM 3: STAND UP FROM CHAIR

**Starting position**	Sitting in chair, bench or plinth with arms folded across chest with his hand up by his shoulders and with feet able to reach floor or supported on secure box. Adjust the seat height so that the hips and knees are at a 90º angle from the floor. Feet no more than shoulder width apart.		
**Instruction**	Can you stand up from the chair keeping your arms folded?		
**Scoring detail**	Score is based on ability to stand up without altering start position.<br>Score 2 - Arms must be kept crossed with the hands up by the shoulders throughout the activity. Feet must remain shoulder width apart.		
**Activity**	2	1	0
**Stand up from chair**	Able to stand up keeping arms folded	With help from thighs / push on chair / prone turn or alters starting position by widening base (moving feet apart)	Unable
**Photographs/ notes**	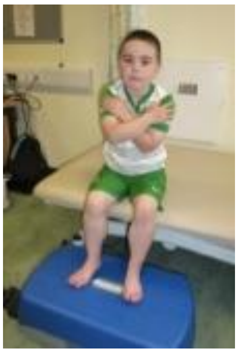<br>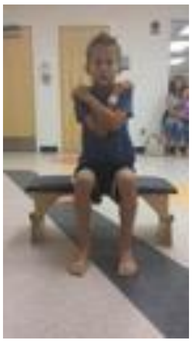<br>Score 2 – keeps arms folded and stands up without widening base of support	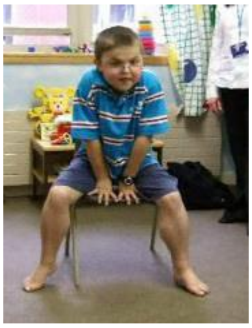<br>Score 1 - Needs two hands to push and widens base of support	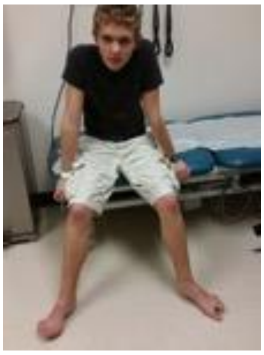<br>Score 0 -Unable to rise from starting position without assistance.

1.8 ITEM 4 & 5: STAND ON ONE LEG RIGHT & LEFT			
**Starting position**	Standing free of support. Near a point of balance if required (evaluator or a bench)		
**Instruction**	"Can you stand on one leg like this?" Count "And 1, and 2, and 3"<br>Demonstrate the task especially if he lifts his leg very high.		
**Scoring detail**	Score is based on ability to stand upright on one leg and the amount of effort to hold this position. Score is not based on arm position. We recommend arms are held at about 45 degrees of abduction but younger children may hold arms higher from a developmental perspective. If they need to hold your hand to lift their foot – you cannot score them from this effort. They must achieve stand on one leg themselves.<br>Score 2 – Minimum count of 3 seconds required in upright posture.<br>Score 1 - A trunk tilt of approximately 20º or more or lifted foot is in contact with weight-bearing leg		
**Activity**	2	1	0
**Stand on one leg**	Able to stand upright in a relaxed manner (no fixation) for a count of 3 seconds	Stands but either momentarily or with trunk side-flexion (20◦) or needs fixation e.g. by thighs adducted	Unable
**Photographs / notes**	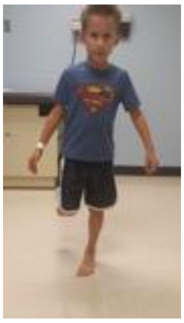<br>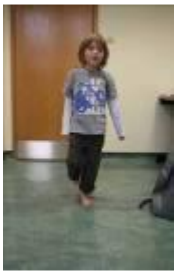<br>Score 2 – Relaxed and no fixation	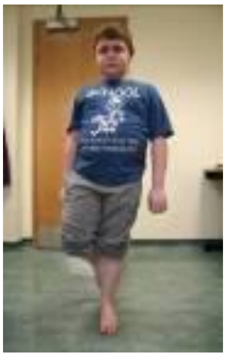<br>Score 1 -Hooks leg behind standing leg<br>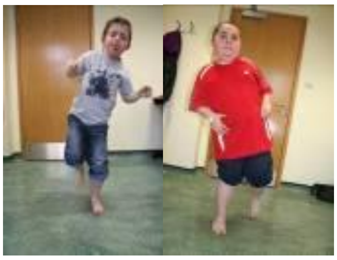<br>Score 1 -Postural compensation / leaning to side at least or more than 20º	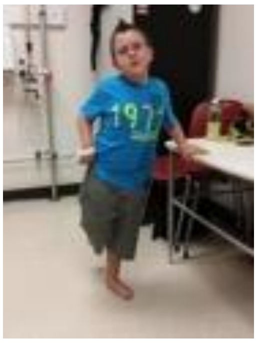<br>Score 0 -If needs to hold onto a table or person for support

1.9 ITEMS 6 & 8: CLIMB BOX STEP RIGHT & LEFT

**Starting position**	Standing in front of box step, toes no more than 20 cm (~8 in) away from box step. Box step must be 15 cm high.		
**Instruction**	Can you step onto the top of the box using your right/ left leg?		
**Scoring detail**	Score is based on ability to get on the step without compensation.<br>Score 1- If they need a hand for balance only – evaluators hand should be kept relatively high or if they use a hand on their thigh. If in doubt whether this is used for balance or support offer them your hand and repeat test.<br>Score 0 - If they put weight through the evaluators hand (with one or two hands) or unable to climb step<br>They should not have their hands in their pocket for a score of 2. Repeat test or counts as compensation and therefore score 1.		
**Activity**	2	1	0
**Climbs box step**	Faces step – no support needed	Goes up sideways / rotates trunk / circumducts hip / needs hands for balance or hands on legs	Unable to perform independently
**Photographs / notes**	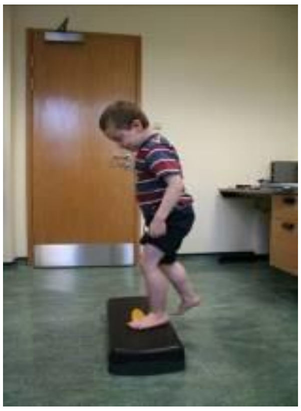	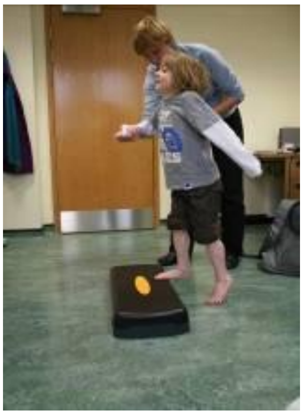<br>Score 1 – Needs hand for balance only	
	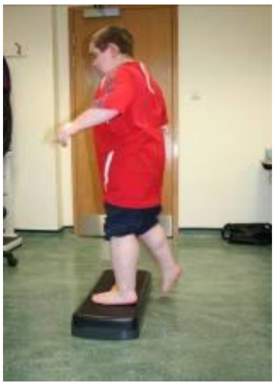<br>Score 2 – Faces step	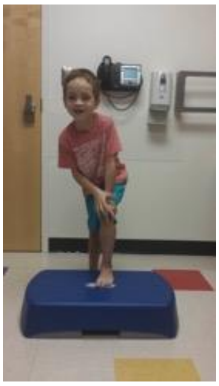<br>Score 1 - Rotates trunk and uses hands on thigh	

1.10 7 & 9: DESCEND BOX STEP RIGHT & LEFT

<table>
<tr><td>Starting position</td><td colspan="3">Standing on box step with feet close to edge but not curling over edge of the step.

Box step must be 15 cm high.</td></tr>
<tr><td>Instruction</td><td colspan="3">Can you step down from the box using your right (or left) leg?

If they rush / skip down, stand in front of the step with space for them to step down and ask them to step down slowly.</td></tr>
<tr><td>Scoring detail</td><td colspan="3">Score is based on ability to get off step without compensation or support

Score 1: If they need a hand for balance only – evaluators hand should be kept relatively high. If they use a hand on their thigh. If in doubt whether this is used for balance or support offer them your hand and repeat test. Uses method that avoids flexing supporting knee (one on the box step)

Score 0: If they put weight through the evaluators hand (with one or two hands) or unable to descend step

They should not have their hands in their pocket. Repeat test or counts as compensation therefore score 1.</td></tr>
<tr><td>Activity</td><td>2</td><td>1</td><td>0</td></tr>
<tr><td>Descends box step</td><td>Faces forward, steps down controlling weight-bearing leg. No support needed</td><td>Sideways / skips down / needs hands for balance or hands on legs</td><td>Unable without more than minimal support, or requires hands for support</td></tr>
<tr><td rowspan="2">Photographs/ notes</td><td>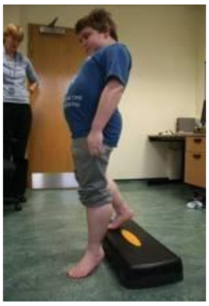
Score 2 – faces forward</td><td>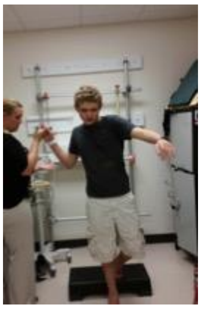
Score 1 – Needs hand for balance. No weight taken through hand.</td><td></td></tr>
<tr><td>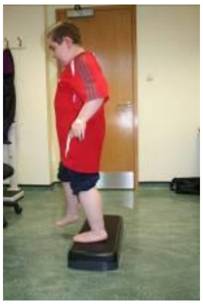</td><td>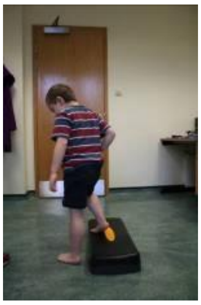
Score 1 – Turning to side to descend</td><td></td></tr>
</table>

1.11 ITEM 10: LIFTS HEAD FROM SUPINE			
**Starting position**	Supine on a plinth or floor, arms folded across chest and the hands up by the shoulders. No pillow must be used.		
**Instruction**	Can you lift your head and touch your chin to your chest keeping your arms folded? If they are not able, demonstrate the action by flexing their neck for them.		
**Scoring detail**	Ask patient to keep arms crossed over chest during the activity to avoid self-assistance.<br>Score 2 – Uses neck flexion to lift head from the beginning of the effort.<br>Score 1 – If they clearly use excessive protraction prior to using neck flexion or only protraction or lacks full range of neck flexion.		
**Activity**	2	1	0
**Lifts head from supine**	In supine, full neck flexion, head must be lifted in mid-line. Chin moves towards chest	Head is lifted through side flexion, partial neck flexion, or with protraction	Unable. No clearance of head from surface
**Photographs / Notes**	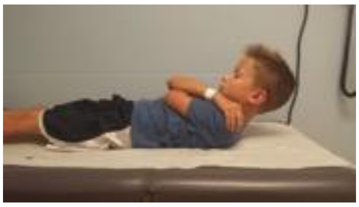<br>Score 2 – Chin on chest using neck flexion.	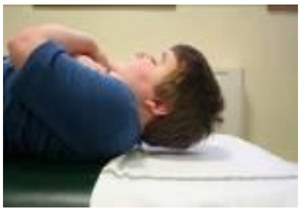<br>Score 1 – Does not achieve full range of flexion, mainly protraction.	

1.12 ITEM 11: GETS TO SITTING			
**Starting position**	Starting position supine on the floor, or large plinth with arms by side. No pillow must be used under head.		
**Instruction**	Can you get from lying into sitting?		
**Scoring detail**	Aim is to move into long sitting rather than sitting over the edge of a plinth. Use of one hand or arm is acceptable to achieve a score of 2.<br>Score 1 - if patient turns into prone or towards the floor to work their way into sitting or if uses two arms. Also score a 1 if the child uses leg momentum/rocking to get to sitting.<br>*They do not sit up so their legs are over the edge of the bed		
**Activity**	2	1	0
**Gets to sitting**	Starts in supine – may use one hand / arm to push up	Uses two arms / pulls on legs or turns towards floor or uses momentum/rocking	Unable
**Photographs /notes**	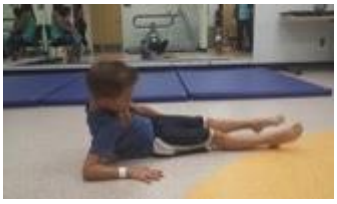 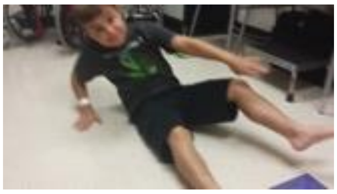 Score 2 - Only needs one arm to sit up	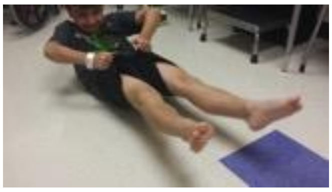 Score 1 - Pulls with both hands pulling on shorts to sit up	

1.13 ITEM 12: RISE FROM FLOOR

**Starting position**	Starting position supine with arms by sides, legs straight. No pillow to be used.		
**Instruction**	• Give the following verbal instructions to the patient:<br>• "When I say GO can you get up as fast as you can and stand up straight with your arms by your side like a soldier using as little support as possible". Give the command "Ready, Set, GO!" and start the stopwatch when saying "GO".<br>• Stop the timer when the patient assumes an upright position with his arms by his side.		
**Scoring detail / Diagram**<br>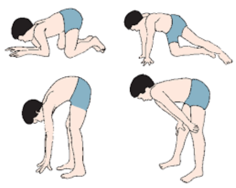	Activity must be attempted without use of furniture in the first instance. Only provide a chair after the patient has attempted to stand from the floor for 30 seconds and failed.<br>Score 0: If the patient touches any object including another person, even if the patient does not use the object for support.<br>Score 1: If the patient gets to standing independently of any furniture but demonstrates any part of the maneuver described below.<br>• Components of Gower's maneuvers:<br>• Turns towards the floor (into a four-point kneeling position or rolls to prone).<br>• Places hands on the floor to assist rising from floor and walks hands back in towards him.<br>• Uses one or both arms to push up on legs to achieve upright standing.<br>• Large base of support by abducting hips and extending knees.<br>Score 1 – If they get up slowly through half kneeling but lean with their chest pointing to the floor. This counts as "prone".		
**Activity**	2	1	0
**Rise from floor**	No evidence of Gower's manoeuvre.	Exhibits at least one of the components described above – in particular rolls towards floor, and/or use hand(s) on legs	(a) NEEDS to use external support object e.g. chair, wall –still record time OR (b) Unable (time must be entered as N/A if the patient scores 0)
**Photographs / Note**	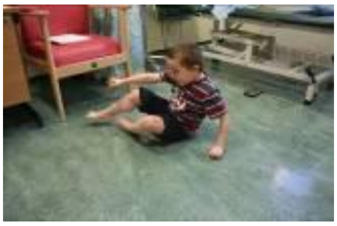<br>Score 2 - Doesn't roll. Only 1 hand on floor to move into sitting	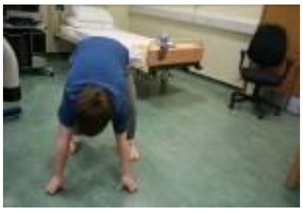<br>Score 1 – Rolls to prone<br>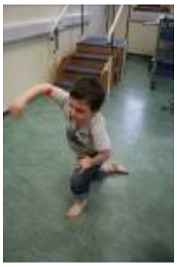<br>Score 1 - Uses hand on thigh	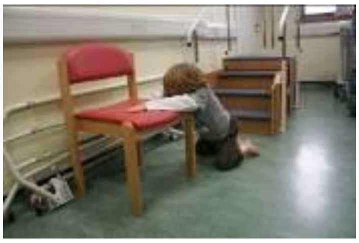<br>Score 0 – Uses chair

**1.14 ITEM 13: STANDS ON HEELS**			
**Starting position**	Standing free of support, arms by side.		
Instruction	Can you stand on your heels? Count 'And 1, and 2, and 3." Helps to demonstrate item.<br>Do not worry how much he sticks his bottom out - does not affect score		
Scoring detail	Score is based on ability to stand on heels for the count of three. It is NOT walking on heels.<br>Score 2: Must clear both feet at the same time using clear dorsiflexion<br>Score 1: If both forefeet are lifted - all metatarsal heads of floor even if inversion is evident or clear dorsiflexion of one foot.<br>Score 0: If uses inversion with lateral border (fifth metatarsal) of foot still on the ground or if just lifts toes.		
Activity	2	1	0
Stand on heels	Both feet at the same time, clearly standing on heels only (acceptable to move a few steps to keep balance) for count of 3	Raises forefoot on both feet – all metatarsal heads off ground – or clearly dorsiflexes one foot only	Unable
Photograph / Notes	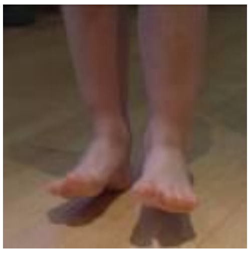<br>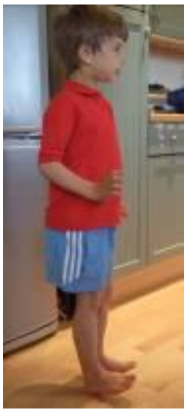<br>Score 2 – Both feet raised	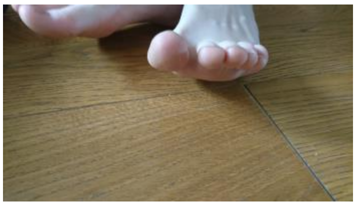<br>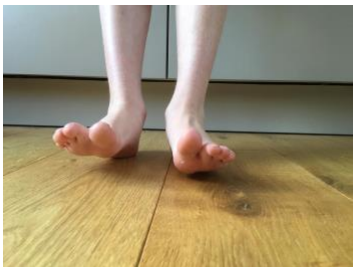<br>Score 1 - Metatarsals off the floor but not clear dorsiflexion of both feet	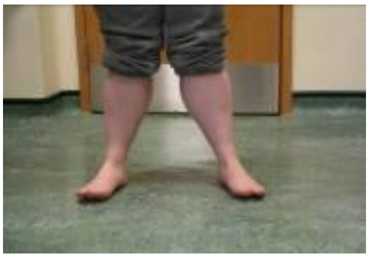<br>Score 0 – Unable to raise even forefeet<br>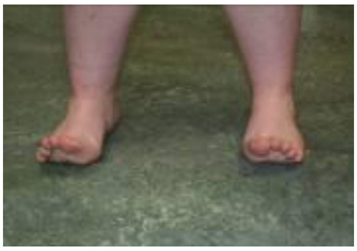<br>Score 0 – Fifth metatarsal head still in contact with floor

1.15 ITEM 14: JUMP

**Starting position**	Standing on the floor, feet comfortably close together		
**Instruction**	Can you jump?		
**Scoring detail**	Score is based on ability to clear the floor with both feet. The item is about height, not jumping forwards although a small amount of forward travel is acceptable.<br>If you hold his hands in order for him to try you cannot score from this attempt.		
**Activity**	2	1	0
**Jump**	Both feet at the same time, clear the ground simultaneously and land at the same time	One foot after the other (skip) or does not fully clear both feet at the same time	Unable
**Photographs / Notes**	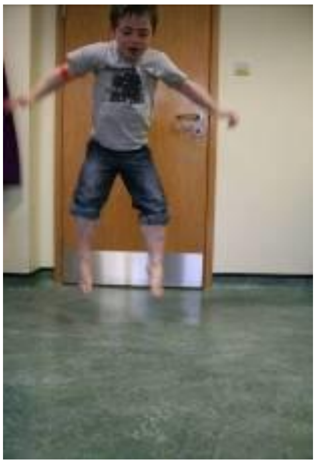<br>Score 2 – Clears floor	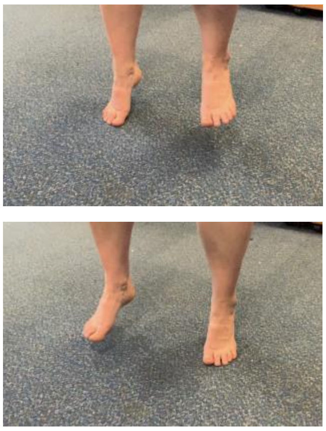<br>Score 1 - One step after the other (skip)	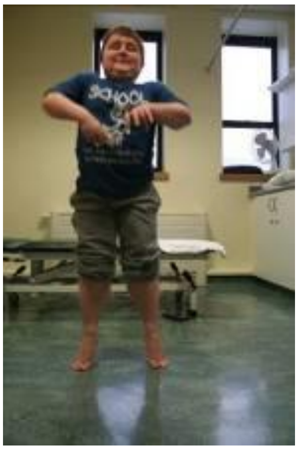<br>Score 0 - Feet do not leave floor

1.16 ITEM 15 & 16: HOP RIGHT & LEFT LEG

**Starting position**	Starting position standing on floor on right / left leg.		
**Instruction**	Can you hop on your right / left leg?		
**Scoring detail**	Score is based on ability to clear the floor. If you hold his hands to attempt this item you cannot score from that attempt.<br>Score 2 - Needs obvious floor clearance. Must leave and land on one foot. If leaves floor but lands with two feet this is not a hop and scores 0.<br>Score 1 – Demonstrates a real effort by bending AND pushing with the knee and ankle at the same time. Real Oomph in the action.<br>Score 0 – If just raises heel or just bends knees.		
**Activity**	2	1	0
**Hop**	Entire foot clears the floor	Able to bend knee AND raise heel, no floor clearance	Unable or only raises heel
**Photographs / Notes**	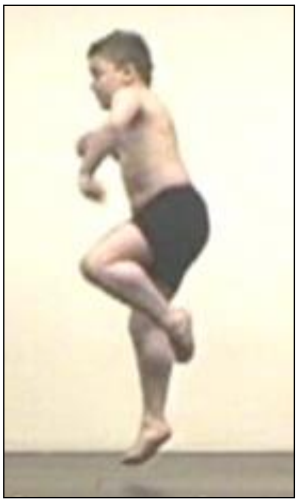<br>Score 2 – Clears foot off floor	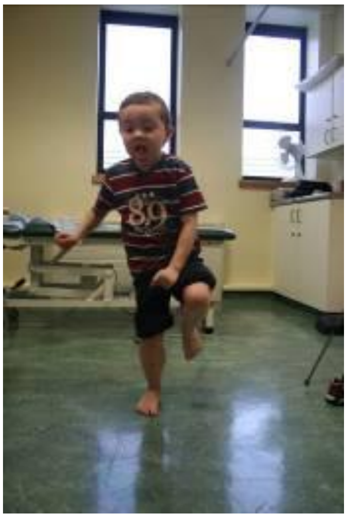	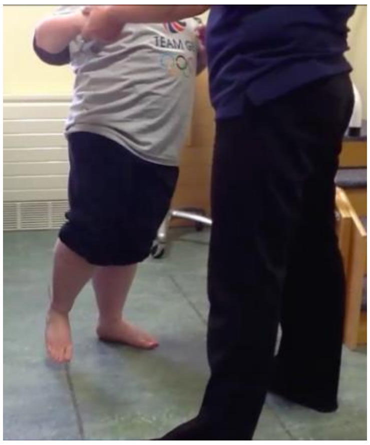
		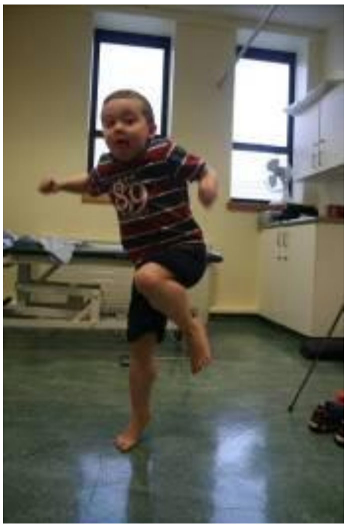<br>Score 1 - Bends knee and raises heel but foot does not leave floor	Score 0

1.17 ITEM 17: RUN (10 METRES)			
Starting position	Standing. Clinical Evaluator to stand 1-2m behind the 10m mark.		
Instruction	Give the following verbal instructions to the patient: **“When I say GO, you go as fast as you safely can all the way to me**. If you can run, then run. If you can’t then walk to me as fast as you can.” (Encourage child to run **past** the 10-metre mark by placing mark or cone at 12m) by standing at least one meter behind the line. Give the command **“Ready, Set – GO”**. Continuously encourage the patient until he crosses the finish line.<br>**Start the timer when you say “GO”.** Stop the timer when the second foot clears the finish line.		
Scoring<br>detail	Score is based on “flight” and speed<br>A straight 12-m walkway must be clearly marked at 10-m in a quiet department or corridor. A stopwatch must be used to time the walk. They must self-select speed after being asked to go ‘as fast as they can’.<br>Score 2 – Clear flight phase – both feet off floor at same time. It may not be normal, as there may be lots of arm movement but a run is achieved.<br>Score 1 – Faster than walking speed for the majority of the 10 metre test. Includes those who ‘Duchenne jog’ - not a true run (There **is** a double support phase), but it is faster than a walk. Characterized by excessive use of arms, trunk rotation, substantial ’waddle’. No real ‘push-off’.<br>Score 0 – Unable to speed up from walking speed or can only speed up briefly.		
Activity	2	1	0
Run	Both feet off the ground (no double stance phase during running)	‘Duchenne jog’ or fast walk	Walk
Photographs / Notes	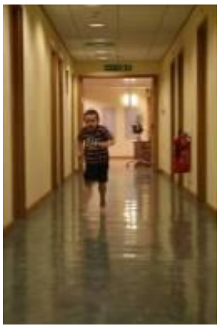<br>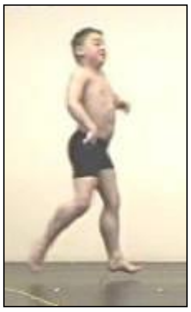<br>Score 2 - Both feet off the ground<br>Definite ‘run’	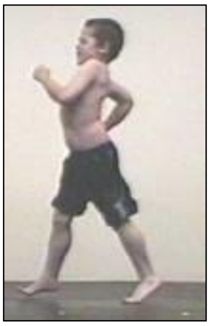<br>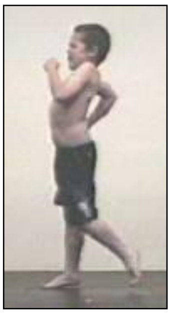<br>Score 1 - Picks up speed but waddles and has one foot in contact with floor at all times	

Score Sheet

Activity	2	1	0	Score
**1. Stand** *3	Stands upright, still, symmetrical, without compensation (heels flat and hips in neutral rotation) for minimum count of 3 seconds	Stands still but with compensation (e.g. on toes or with legs abducted or with bottom stuck out/hip flexion, etc.) for minimum count of 3 seconds	Cannot stand still or cannot stand independently, needs support (even minimal)	
**2. Walk** *3	Walks consistently with heel-toe or flat-footed gait pattern	Persistent or habitual toe walker, unable to heel-toe consistently	Loss of independent ambulation – may use knee-ankle-foot orthosis (KAFO) or walk short distances with assistance	
**3. Stand up from chair** *3	Able to stand up keeping arms folded	With help from thighs / push on chair / prone turn or alters starting position by widening base (moving feet apart)	Unable	
**4. Stand on one leg – right** *3.5	Able to stand upright in a relaxed manner (no fixation) for a count of 3 seconds	Stands but either momentarily or with trunk side-flexion (20◦) or needs fixation e.g. by thighs adducted	Unable	
**5. Stand on one leg – left** *3.5	Able to stand upright in a relaxed manner (no fixation) for a count of 3 seconds	Stands but either momentarily or with trunk side-flexion (20◦) or needs fixation e.g. by thighs adducted	Unable	
**6. Climb box step – right** *3	Faces step – no support needed	Goes up sideways / rotates trunk / circumducts hip / needs hands for balance or hands on legs	Unable to perform independently	
**7. Descend box step – right** *3.5	Faces forward, steps down controlling weight-bearing leg. No support needed	Sideways / skips down / needs hands for balance or hands on legs	Unable without more than minimal support, or requires hands for support	
**8. Climb box step – left** *3	Faces step – no support needed	Goes up sideways / rotates trunk / circumducts hip / needs hands for balance or hands on legs	Unable to perform independently	
**9. Descend box step -left** *3.5	Faces forward, steps down controlling weight-bearing leg. No support needed	Sideways / skips down / needs hands for balance or hands on legs	Unable without more than minimal support, or requires hands for support	
**10. Lifts head** *4	In supine, full neck flexion, head must be lifted in mid-line. Chin moves towards chest	Head is lifted through side flexion, partial neck flexion, or with protraction	Unable. No clearance of head from surface	
**11. Gets to sitting** *3	Starts in supine – may use one hand / arm to push up	Uses two arms / pulls on legs or turns towards floor or uses momentum/rocking	Unable	
**12. Rise from floor** *4	No evidence of Gower's manoeuvre.	Exhibits at least one of the components described above – in particular rolls towards floor, and/or use hand(s) on legs	(a) NEEDS to use external support object e.g. chair, wall – still record time OR (b) Unable (time must be entered as N/A if the patient scores 0)	
**13. Stands on heels** *3.5	Both feet at the same time, clearly standing on heels only (acceptable to move a few steps to keep balance) for count of 3	Raises forefoot on both feet – all metatarsal heads off ground – or clearly dorsiflexes one foot only	Unable	
**14. Jump** *3	Both feet at the same time, clear the ground simultaneously and land at the same time	One foot after the other (skip) or does not fully clear both feet at the same time	Unable	
**15. Hop right leg** *4	Entire foot clears the floor	Able to bend knee AND raise heel, no floor clearance	Unable or only raises heel	
**16. Hop left leg** *4	Entire foot clears the floor	Able to bend knee AND raise heel, no floor clearance	Unable or only raises heel	
**17. Walk Run** (10 m) *3	Both feet off the ground (no double stance phase during running)	'Duchenne jog' or fast walk	Walk	
			**TOTAL=**	**/34**

Timed RFF: no time if uses furniture _____.___ **Timed 10m run / walk** _____.___

Age at which 85% of controls achieve full score *3 = 3 years of age, *3.5 = 3.5 years of age, *4 = 4 years of age (Mercuri 2016)
