# Supplementary material for: Determining minimal clinically important differences in the North Star Ambulatory Assessment (NSAA) for patients with Duchenne muscular dystrophy
Source: PLoS One. 2023 Apr 26;18(4):e0283669. doi: 10.1371/journal.pone.0283669 (PMC10132589; doi:10.1371/journal.pone.0283669)
Supplement: Attachment — Submitted filename: Response to Reviewers Jan 2023.docx [file pone.0283669.s004.docx]

PONE-D-22-17578

Determining minimal clinically important differences in the North Star Ambulatory Assessment (NSAA) for patients with Duchenne muscular dystrophy.

PLOS ONE

Dear Dr. Alway,

Thank you for giving us the opportunity to address further comments from the reviewer for our manuscript “Determining minimal clinically important differences in the North Star Ambulatory Assessment (NSAA) for patients with Duchenne muscular dystrophy” for publication.

We are greatly appreciative of your and the reviewers’ time and efforts to provide us feedback and improve our manuscript. We have carefully considered the comments and have tried our best to address each of them. We hope the manuscript after careful revisions meet your standards for publication.

Please see below, a point-by-point response to the reviewer’s comments. All modifications in the manuscript are highlighted as track changes in the file labelled 'Revised Manuscript with Track Changes'. Page and line numbers in the responses refer to this file. In line with the suggested changes, any changes to the references are as highlighted in the track changes. We also provide a revised Figure 1 to address comment 3.

**Reponses to reviewer comments**

1. *Reviewer #2: Most of my comments have been addressed. I did have some additional comments on the revision. In the new Table 1, the range for the NSAA score is listed as 2-34. Please verify that the low score of 2 is correct. Given that the oldest participant in the cohort is listed as being 10.5 years old, this would be an unexpectedly low NSAA score for a group of Duchenne patients this age.*

Author response: Previous and recent data checks did not find any errors in the study dataset. While this is not necessarily typical, given that average age of loss of ambulation in DMD patients is 12 years, a score of 2 at the age of 10.5 years is plausible and can be expected in a large cohort. Indeed, our previous studies evaluating large cohort in DMD do not contradict this finding (see NSAA distribution scores and loss of ambulation in the large cohort study published in 2019: Muntoni F, Domingos J, Manzur AY, et al. Categorising trajectories and individual item changes of the North Star Ambulatory Assessment in patients with Duchenne muscular dystrophy. PLoS One. 2019 Sep 3;14(9):e0221097. doi: 10.1371/journal.pone.0221097. PMID: 31479456; PMCID: PMC6719875)

2. *Reviewer #2: It was not apparent in the original manuscript that only 31 participants were included in the distribution-based MCID analysis of the six-minute walk test. Since the original cohort from the IMDEX natural history study had 56 participants, only about half of the cohort is represented in this analysis. This rather alters my perception of the robustness of this analysis. It would be useful to get a better understanding of the reasons that so much of the data is missing from this cohort. Has there been any analysis to determine how representative these 31 participants are with respect to the larger cohort being described here? Is there any reason to believe that participants were excluded for reasons relating to their physical function?*

Author response: The study data is limited in six-minute walk distance data (this had been noted within study limitations). On evaluating characteristics across the overall IMDEX sample (N=56) and the six minute walk distance sample in the study (N= 31), we found the smaller cohort of 31 patients with six minute walk distance data available to be representative of the overall IMDEX sample in the study, and thus did not find evidence of systematic bias relating to physical function (please see the table attached below).

|  | Overall IMDEX cohort (N=56) | Cohort with 6MWD available (N=31) |
| --- | --- | --- |
| Age, mean (sd) | 7.6 (2.5) | 7.6 (1.1) |
| NSAA at first visit, mean (SD), range | 22.8 (7.8), 7-34 | 25.0 (7.6), 8-34 |
| % on steroids | 96% | 97% |
| % on daily steroids | 62% | 60% |
| % on intermittent steroids | 32% | 33% |
| % unknown regime | 6% | 7% |

We have added the sentence below in the methods section (lines 182-184) to indicate representativeness of the six-minute walk distance sample.

“The patient characteristics of the cohort for whom the 6MWD data was available for this study was representative of patient characteristics in the wider iMDEX natural history cohort.”

3. *Reviewer #2: Results: “Based on 62 observed annual changes in 24 boys (54 observations) above the ages of seven years….” Please clarify how the 62 changes were counted from 54 observations. If the 24 participants were each measured at baseline and after 1 year, that would be 48 observations. If 6 of those participants had an additional measurement after year 2, that would make 54 observations, but this would only end up being 30 annual changes, not 62. There also appear to be more than 62 points plotted in Figure 1, which is supposed to correspond to this analysis. If participants are contributing multiple data points to the analysis, please describe any adjustment for non-independent data used in the correlation analysis.*

Author response: Thank you for this comment. We agree that this section appears inconsistent and requires clarification. To address this and account for multiple changes per patient, we have made the following changes to the manuscript:

Lines 312-316 have been revised to:

“Based on 36 non-overlapping observed annual changes in 24 boys above the ages of 7 years, a positive correlation of 0.50 (p=0.002) was observed between change in NSAA and change in 6MWD (Fig 1). Using mixed methods regression analysis to account for multiple changes per patient and the previously reported MCID for 6MWD as a decline of 30m^17^, the equivalent decline in NSAA was observed as -3.5 points (95% CI= -1.9, -5.0).”

In addition, Figure 1 has been revised to display non-overlapping changes (attached below). MCID for NSAA by the anchor method has been corrected to 3.5 (previously noted at 3.3) in the abstract and discussion.
